# Ureteroinguinal Hernia: A Rare General Surgery Phenomenon

**DOI:** 10.7759/cureus.20586

**Published:** 2021-12-21

**Authors:** Alexandria Turner, Pradeep Subramanian

**Affiliations:** 1 General Surgery, Darling Downs Health Service, Toowoomba, AUS

**Keywords:** symptomatic hernia, recurrent inguinal hernia, general and laparoscopic surgery, inguinal hernia surgery, ureteral inguinal hernia

## Abstract

An inguinal hernia involving the ureter is extremely rare and even rarer in patients with native kidneys. It can occur with or without obstructive uropathy and as such, a high index of suspicion should be held for patients with urinary symptoms and concurrent inguinal hernia. Additionally, iatrogenic ureter injury can occur if surgeons are unaware of ureteral involvement pre-operatively. We present a case of a patient with a known ureteroinguinal hernia who proceeded to have an elective hernia repair with ureteral protection.

## Introduction

Ureteroinguinal hernias are an extremely rare phenomenon with less than 140 cases documented since 1880; most cases are identified intraoperatively [1-7]. Risk factors for developing ureteroinguinal hernia include male sex, increased age and history of renal transplant; such hernias can occur with or without urinary symptoms. Preoperative signs and symptoms include unexplained renal failure, lower urinary tract symptoms (LUTS), and unilateral hydronephrosis on imaging [5]. 

We report on a symptomatic, native kidney patient who was diagnosed with a ureteroinguinal hernia preoperatively.

## Case presentation

A 76-year-old man was referred to the General Surgery clinic by our Urology colleagues with a left indirect ureteroinguinal hernia. Past medical history was significant for renal colic, atrial fibrillation, ischemic heart disease, permanent pacemaker, Ivor-Lewis oesophagectomy for Barrett’s oesophagus with high-grade dysplasia, open right inguinal hernia repair and open umbilical hernia repair.

He was initially seen by our urology colleagues five months earlier, after being referred by his GP with LUTS. He reported a three-month history of occasional urgency, nocturia, weak flow and some terminal dribbling. Digital rectal examination (DRE) was insignificant with a benign prostate. An ultrasound of kidneys, ureter and bladder (USS KUB) showed a 48cc prostate with slight prominence of the left renal pelvis with no obstructing cause identified. He was subsequently commenced on Duodart (dutasteride 500 mcg, tamsulosin [controlled release] 400 mcg) daily.

**Figure 1:**
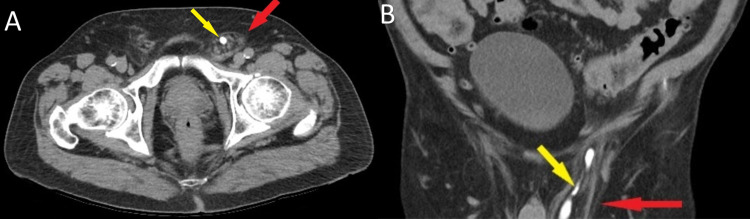
Preoperative CT scan of the abdomen and pelvis (A, B) Left inguinal hernia (red arrow) containing ureter (yellow arrow)

On review two months later, he reported a few episodes of painless macroscopic haematuria and had a computed tomography intravenous pyelography (CT IVP) to exclude renal stone. This CT revealed that the distal aspect of the left ureter extended into the left inguinal canal within a left indirect inguinal hernia. The kidneys and renal tracts were otherwise unremarkable apart from simple-appearing renal cysts, with no stone disease or obstructive uropathy (Figures 1A, 1B). The patient's serum and urinary chemistry values were normal.

**Figure 2:**
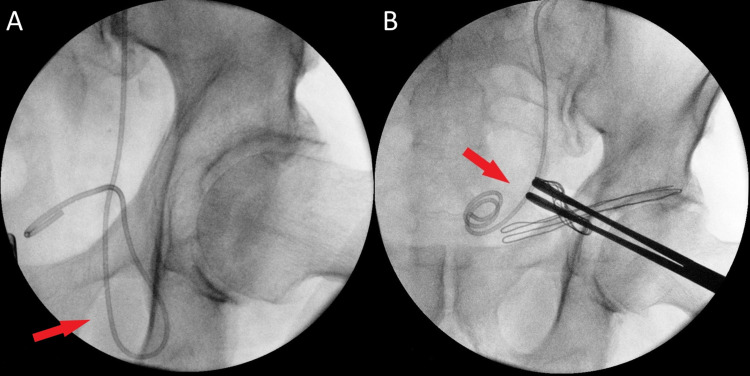
Preoperative and intraoperative retrograde pyelogram (A) Ureter (red arrow) descending into the left inguinal hernia. (B) Ureter (red arrow) completely reduced.

**Figure 3:**
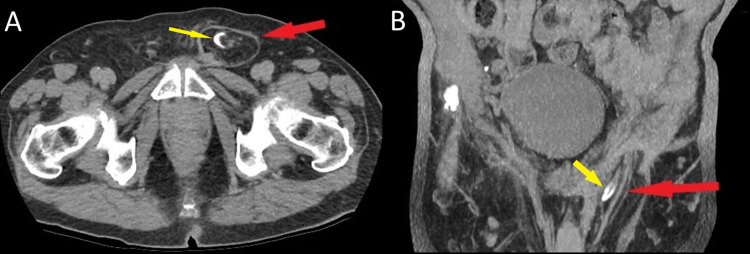
Preoperative CT scan of the abdomen and pelvis (A, B) Recurrence of left inguinal hernia (red arrow) containing ureter (yellow arrow)

**Figure 4:**
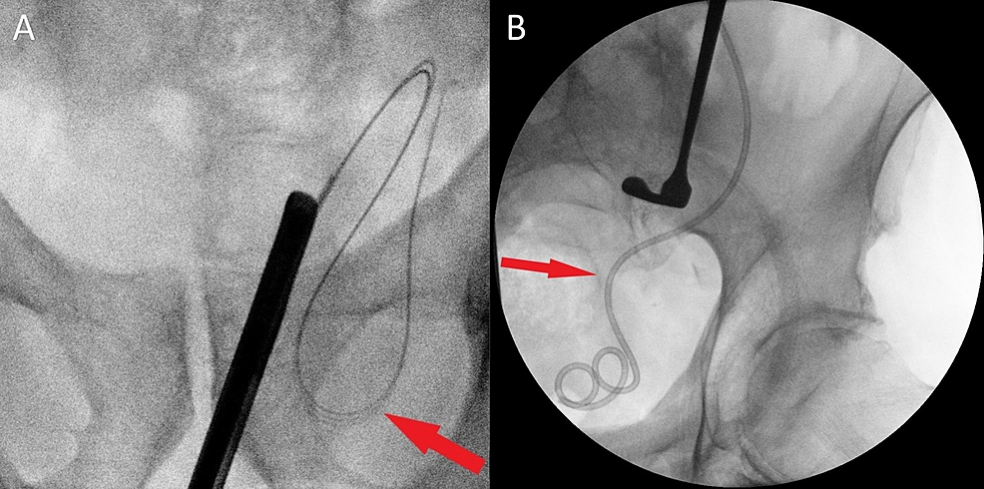
Preoperative and intraoperative retrograde pyelogram (A) Ureter (red arrow) descending into the left inguinal hernia. (B) Ureter (red arrow) completely reduced.

The patient was consented to and underwent pre-operative insertion of a left ureteric stent and an open left tension-free inguinal hernia repair with Ultrapro mesh (Figure 2A). Intraoperative imaging was used to confirm the intra-abdominal position of the ureter after the reduction of hernia (Figure 2B). The patient remained an inpatient for two days. Unfortunately, the hernia reoccurred after the patient lifted wood approximately seven weeks postoperatively. A CT IVP was ordered which again showed a left ureteroinguinal hernia with a long redundant segment within the left retroperitoneal fat extending into the left inguinal canal (Figures 3A, 3B). The patient underwent insertion of left ureteric stent preoperatively and a redo open Rives-Stoppa left inguinal hernia repair with Progrip mesh (Figure 4A). Intraoperative imaging was used to confirm that the left ureteric stent was located retroperitoneally post-reduction of hernia (Figure 4B). The patient remained an inpatient for three days and had an uneventful recovery with nil further recurrence of hernia at the six-month postoperative review.

## Discussion

Inguinal hernia repairs are one of the most common medical conditions encountered by general surgeons, with more than 20 million inguinal hernia repairs being undertaken worldwide [6]. Ureteral involvement remains rare, but when present is associated with inguinal hernia twice as often as femoral hernias [5,6]. Both sexes are affected, with inguinal hernia predominating in men and femoral hernias in female patients [6]. When an ureteroinguinal hernia is present, they are mostly indirect rather than direct and occur more frequently on the right than the left side [5,6]. Risk factors for developing a ureteroinguinal hernia include male gender, increased age with most hernias occurring after the fifth decade of life and obesity [4]. Patients with a history of kidney transplants also have increased risk, possibly due to the location of the transplanted ureter more anteriorly in the space of Retzius [4,5,7].

Ureteroinguinal is classified as either paraperitoneal or extraperitoneal, based on the presence or absence of a hernial sac, respectively [3,5,6]. The paraperitoneal type is more common, associated with a hernial sac, and accounts for 80% of cases. It is a sliding hernia and may develop due to traction on the ureter by abnormally adherent posterior parietal peritoneum. The ureter, as a retroperitoneal structure, is involved as part of the hernia sac wall, rather than as an internal component of the hernia sac [3-6].

The extraperitoneal hernia is not associated with a hernia sac and results from an embryological abnormality in which the ureteric bud separates late from the Wolffian duct as it descends to form the epididymis and testis [4-6]. The ureter without the peritoneum attached and a large amount of retroperitoneal fat herniates into the inguinal canal [4,5]. This was the case with our patient. 

Preoperative diagnosis of ureteral hernia is optimal to avoid inadvertent ureteral injury; however, routine preoperative workup of inguinal hernia does not require any specific imaging. Signs that warrant further investigation include flank pain, dysuria, haematuria, acute urinary obstruction, double voiding requiring pressure to initiate or terminate voiding [4,6]. Further evaluation with cross-sectional imaging with a non-contrast CT allows assessment of any renal obstruction and anatomical classification of any hernias identified [8]. While ultra-low-dose scanning protocols allow nearly equivalent radiation doses as an abdominal x-ray, CT scans should be limited to patients in whom there is a clinical suspicion of pathology and whose management will be altered based on results [4,8].

Herniorrhaphy is indicated in all cases of ureteroinguinal hernias to prevent obstructive uropathy, incarceration or strangulation of the hernia and can be an open or laparoscopic procedure [4,7,8]. If ureter involvement is identified preoperatively, urethral protection with a ureteric stent preoperatively allows identification of and protection of the ureter during hernia repair as was the case for our patient [4,7,8].

Surgeons should be cautious when they encounter a hernia that contains fat that does not meet the characteristics of a cord lipoma and lacks the presence of a hernia sac. Careful dissection of fat from cord structures, and reduction to the retroperitoneum, rather than ligating and excising fat, should be performed to avoid iatrogenic ureteral injuries [5,7]. If unable to completely reduce hernia contents, careful dissection at the level of the deep ring should be carried out to avoid iatrogenic ureteral injury [7].

## Conclusions

While ureteroinguinal hernias are rare, general surgeons need to be aware of these types of hernias so as to avoid intra-operative iatrogenic damage to the ureter which can have serious consequences. There should be an increased emphasis on preoperative diagnosis - a urological history should be taken in patients presenting with an inguinal hernia to guide further investigations. When ureteroinguinal hernia is identified preoperatively, a multidisciplinary approach between general surgery and urology should be undertaken to ensure the best outcome for the patient.
